# Delay of EGF-Stimulated EGFR Degradation in Myotonic Dystrophy Type 1 (DM1)

**DOI:** 10.3390/cells11193018

**Published:** 2022-09-27

**Authors:** Eva Alegre-Cortés, Alberto Giménez-Bejarano, Elisabet Uribe-Carretero, Marta Paredes-Barquero, André R. A. Marques, Mafalda Lopes-da-Silva, Otília V. Vieira, Saray Canales-Cortés, Pedro J. Camello, Guadalupe Martínez-Chacón, Ana Aiastui, Roberto Fernández-Torrón, Adolfo López de Munain, Patricia Gomez-Suaga, Mireia Niso-Santano, Rosa A. González-Polo, José M. Fuentes, Sokhna M. S. Yakhine-Diop

**Affiliations:** 1 Departamento de Bioquímica y Biología Molecular y Genética, Facultad de Enfermería y Terapia Ocupacional, Universidad de Extremadura, Avda de la universidad s/n, 10003 Cáceres, Spain; 2 Instituto de Investigación Biosanitaria de Extremadura (INUBE), 06071 Cáceres, Spain; 3 Centro de Investigación Biomédica en Red de Enfermedades (CIBERNED), 28031 Madrid, Spain; 4 iNOVA4Health, NOVA Medical School|Faculdade de Ciências Médicas, NMS|FCM, Universidade Nova de Lisboa, 1169-056 Lisboa, Portugal; 5 Departamento de Fisiología, Facultad de Veterinaria, Universidad de Extremadura, 10003 Cáceres, Spain; 6 Instituto Universitario de Biomarcadores de Patologías Metabólicas, 10003 Cáceres, Spain; 7 Cell Culture Platform, Biodonostia Health Research Institute, 20014 San Sebastián, Spain; 8 Neuroscience Area of Biodonostia Health Research Institute, Donostia University Hospital, 20014 San Sebastián, Spain; 9 Department of Neurology, Donostia University Hospital, 20014 Osakidetza, Spain; 10 Ilundain Foundation, 20014 San Sebastian, Spain; 11 Department of Neurosciences, University of the Basque Country UPV-EHU, 48940 San Sebastián, Spain

**Keywords:** AKT, autophagy, DMPK, endosomes, LBPA, lysosomes, muscle atrophy

## Abstract

Myotonic dystrophy type 1 (DM1) is an autosomal dominant disease caused by a CTG repeat expansion in the 3′ untranslated region of the dystrophia myotonica protein kinase gene. AKT dephosphorylation and autophagy are associated with DM1. Autophagy has been widely studied in DM1, although the endocytic pathway has not. AKT has a critical role in endocytosis, and its phosphorylation is mediated by the activation of tyrosine kinase receptors, such as epidermal growth factor receptor (EGFR). EGF-activated EGFR triggers the internalization and degradation of ligand–receptor complexes that serve as a PI3K/AKT signaling platform. Here, we used primary fibroblasts from healthy subjects and DM1 patients. DM1-derived fibroblasts showed increased autophagy flux, with enlarged endosomes and lysosomes. Thereafter, cells were stimulated with a high concentration of EGF to promote EGFR internalization and degradation. Interestingly, EGF binding to EGFR was reduced in DM1 cells and EGFR internalization was also slowed during the early steps of endocytosis. However, EGF-activated EGFR enhanced AKT and ERK1/2 phosphorylation levels in the DM1-derived fibroblasts. Therefore, there was a delay in EGF-stimulated EGFR endocytosis in DM1 cells; this alteration might be due to the decrease in the binding of EGF to EGFR, and not to a decrease in AKT phosphorylation.

## 1. Introduction

Myotonic dystrophy type 1 (DM1) is an inherited disease characterized by progressive muscle weakness and wasting. DM1 is the most common muscular dystrophy; its prevalence varies between 1 and 35 per 100,000 people [1,2]. It is an autosomal dominant disease caused by a nucleotide repeat expansion of cytosine–thymine–guanine (CTG) in the 3′ untranslated region (UTR) of the *dystrophia myotonica protein kinase* (*DMPK*) gene [1,3]. Unaffected individuals have 5–37 CTG repeats that remain stable over generations, whereas DM1 patients display more than 40 CTG repeats that tend to increase in successive generations [3]. DM1 is a multisystemic disorder that affects many organs and tissues and can result in endocrine system dysfunction, cataracts, respiratory failure, cancer, and cardiac defects, leading to sudden death in many cases [2,4]. Depending on the onset of the first clinical symptoms, DM1 is classified into five clinical forms: congenital (neonatal), infantile (1 month–10 years), juvenile (11–20 years), adult (21–40 years), and late-onset (40 years and older). Ninety-one percent of congenital forms and longer CTG repeats are associated with maternal inheritance. However, although severe symptoms have been related to maternal inheritance, they only represent 37% of DM1 patients [3]. The severity of muscle atrophy and wasting is positively correlated with the CTG expansion length. The CTG expansion length is related to the induction of cell death and autophagy in various DM1 models such as in the *Drosophila* DM1 (480 CTG) model [5], human DM1 neural stem cells (1000 CTG) [6], and human DM1 myotubes (90–1800 CTG) [7].

Autophagy is a catabolic mechanism that removes toxic proteins and damaged organelles; its impairment, and especially its exacerbation, have been associated with muscle atrophy in a DM1 mouse model [8]. This cellular process is altered in several DM1 models, such as in *Drosophila* [5], human skeletal actin (HSA) ^LR^ mice, and human muscle cells [9], due to deregulation of the mammalian target of rapamycin (mTOR)/AKT [6,7] and AMP-activated protein kinase (AMPK) pathways, which alter a suitable cellular response upon starvation in DM1 mice [9]. DM1 animals exhibiting severe muscle loss displayed increased AMPK activity and phosphatidylinositol 3-kinase (PI3K)/AKT pathway deregulation [8]. AKT acts upstream of mTORC1, and its decrease in phosphorylation (p-AKT) activates autophagy. Moreover, a decrease in AKT activity (p-AKT) is associated with muscle atrophy [10]. There are numerous discrepancies related to AKT/mTOR pathway regulation in DM1. On the one hand, muscle biopsies from DM1 patients [9] and human DM1 neural stem cells (NSCs) [6] show no significant changes in phosphorylated AKT (Ser473), but the mTOR downstream target, phosphorylated ribosomal protein S6, is decreased [6]. On the other hand, under starvation conditions, there is a decrease in AKT activity in DM1, while mTOR is not affected [9].

AKT signaling is regulated by endocytosis through the sorting of epidermal growth factor receptor (EGFR) towards degradation or recycling pathway [11]. The activation of EGFR upon EGF stimulation is sufficient to induce AKT phosphorylation (Thr308/Ser473) [12], which, in turn, leads to the activation of mTOR to promote protein synthesis [10]. Endocytosed receptors from the plasma membrane are conducted to early endosomes, where they can either be recycled to the cell surface or be transferred into late endosomes and then lysosomes for degradation [13]. The internalization of EGFR and its degradation in lysosomes attenuate receptor signaling, and therefore, the PI3K/AKT pathway [14]. Receptor recycling restores and maintains this signal [11] as well as signals of the downstream substrates (AKT/ERK) [15]. An inhibitor of AKT, AKTVIII has been reported to reduce EGFR degradation, but not completely. This inhibition is thought to reduce EGFR recycling [12]. It was previously reported that AKT phosphorylation is decreased in DM1-derived fibroblasts [16]. However, there are no data on the regulation of the endocytic pathway in DM1.

In this study, we characterized the endosomal–lysosomal pathway in primary fibroblasts from healthy subjects (control) and DM1 patients. We observed that the size of endosomes and lysosomes were significantly enhanced in DM1 cells as compared with control (CTRL) cells. Thereafter, we stimulated primary fibroblasts from DM1 patients with a high concentration of EGF to promote EGFR internalization and subsequent lysosomal degradation of EGFR. EGF-stimulated DM1 cells showed a significant decrease in EGF binding and EGFR trafficking during the early steps of endocytosis. However, EGF-stimulated DM1 cells displayed an increase in the phosphorylation levels of AKT and ERK1/2 followed by a phase of decay, excluding an inhibition of the EGFR signaling. Therefore, there is a delay in EGF-stimulated EGFR endocytosis, suggesting an altered intracellular trafficking in DM1 cells.

## 2. Materials and Methods

### 2.1. Cell Culture

Human fibroblasts (HFs) from skin biopsies were obtained from three healthy individuals (38–69 years old) and from three myotonic dystrophy type 1 (DM1) patients with 833, 333, or 500 CTG repeats (41–57 years old). Isolation of primary fibroblasts was performed as previously reported [16]. The experiments were performed in agreement with the *Comité Ético de Investigación Clínica del Área Sanitaria de Gipuzkoa* and with the written and informed consent of the participating subjects in accordance with the Declaration of Helsinki. The cells were maintained in Dulbecco’s modified Eagle’s medium (DMEM, Sigma-Aldrich, Merck KGaA, Darmstadt, Germany, D6546) supplemented with 1% L-glutamine (Sigma-Aldrich, G7513), 10 U/mL penicillin/100 µg/mL streptomycin (Gibco, Grand Island, NY, USA, 15140-122), and 10% fetal bovine serum (FBS, Sigma-Aldrich, F7524) [17]. In the experiments, HFs were seeded between 35,000 and 40,000 cells/mL.

### 2.2. Treatments

Cells were seeded in 6-well plates 24 h prior to any treatment with the following compounds: bafilomycin A1 (BAF.A1, LC Laboratories, Woburn, MA, USA, R-5000, B-1080, 100 nM) [18]; rapamycin (RAPA, Fisher, Pittsburgh, PA, USA, BP2963.1, 1 µΜ) [19], Hegf (E9644, 100 ng/Ml), pepstatin A (P5318, 50–100 µM), DMSO as a vehicle (dilution factor 1:1000), all from Sigma-Aldrich; and leupeptin (Enzo, ALX-260–009, Farmingdale, NY, USA, 50–100 µM).

### 2.3. Western Blot Analysis

To analyze the protein expression levels, cell lysis was performed in buffer containing 0.5% Nonidet P40 (Roche, Mannheim, Germany, 11754599001), 100 mM Tris-HCl (pH 7.4), 300 mM NaCl, protease, and phosphatase inhibitors [20,21]. Proteins were resolved by SDS-gel electrophoresis, and the blots were probed with the following antibodies: HRP-β-actin (AC-15) (ab49900, 1:20,000), cathepsin B (CTSB, ab58802, 1:1000), and LAMP1 (#24170, 1:1000) from Abcam, Cambridge, UK; CTSB (H-5) (sc-365558, 1:1000), CTSC (D-6) (sc-74590, 1:1000), CTSD (D-7) (sc-377299, 1:1000), LAMP1 (H4A3) (sc-20011, 1:1000) and LAMP2 (H4B4) (sc-18822, 1:1000) from Santa Cruz Biotechnology, Dallas, DX, USA; EEA1 (#2411, 1:1000), EGFR (D38B1) (#4267T, 1:1000), Rab5A (#2143, 1:1000), Rab7 (D95F2) (#9367, 1:1000), phosphor-AKT (ser473, #9271), phosphor-ERK1/2(Thr202/Tyr204, #9101), phosphor-mTOR (ser2448, #2971, 1:1000), phosphor-S6 ribosomal protein (D57.2.2E) (ser235/236, #4858, 1:2000), α-tubulin (DM1A) (TUBB, #3873S, 1:1000) from Cell Signaling Technology, Danvers, MA, USA; GAPDH (MAB374, 1:5000) from Merk-Millipore, Burlington, MA, USA; CTSL (CPL33/1) (C4618, 1:1000) and LC3 (L7543, 1:5000) from Sigma-Aldrich; and SQSTM1/p62 (#H00008878, 1:5000) from Abnova, Taibei, China.

### 2.4. Immunofluorescence Microscopy

Cells were fixed with 4% paraformaldehyde (PFA) and permeabilized with either 0.1% Triton X-100 (Sigma-Aldrich, T9284) in PBS for 5 min [20] or 0.01% saponin (Fluka BioChemika, Merck KGaA, Darmstadt, Germany, #47036) in BSA (1 mg/mL) for one hour. Saponin was only used for endocytosis/lysosome labeling. Triton permeabilization was followed by a one-hour incubation in BSA. After permeabilization, the cells were incubated with primary antibodies against EEA1 (C45B10) (Cell Signaling Technology, Danvers, MA, USA, #3288P, 1:100), EGFR (D38B1) (Cell Signaling Technology #4267S, 1:100), LAMP1 (H4A3) (sc-20011, 1:200), and SQSTM1 (D-1) (sc-28359, 1:200) overnight at 4 °C or for one hour at RT. The cells were then incubated with Alexa Fluor^®^ 568 (A11004)- or 488 (A11008)-conjugated secondary antibodies (ThermoFisher, Waltham, MA, USA) for 1 h at RT. Nuclei were stained with 4′,6-diamidino-2-phenylindole (DAPI [300 nM], Invitrogen D1306) [20]. Images were visualized using an Olympus IX51 inverted microscope equipped with a DP71 camera and with a confocal microscope (A1 confocal imaging system mounted on an inverted Eclipse Ti microscope (Nikon Corp., Tokyo, Japan). The scale of 8-bit images was set from pixels to microns, the threshold was adjusted, and the particle circularity was fixed between 0 and 1. The particle number and the average area occupied in µm^2^ by those particles were analyzed with ImageJ software 1.53f51, National Instiutes of Health, USA.

### 2.5. EGFR Degradation

The cells were seeded in 6-well plates containing complete DMEM (as described above). The following day, HFs were washed with PBS and starved overnight in FBS-free DMEM supplemented with 0.1% BSA and L-glutamine (termed binding DMEM) [22]. The cells were then treated with or without hEGF (100 ng/mL) in binding DMEM for 0, 15, 30, or 60 min at 37 °C to monitor EGFR internalization and degradation. Depending on the aim of the experiment, the cells were pretreated with leupeptin or BAF.A1. To stop cell treatment, the plates were washed with ice-cold PBS and immediately stored at −80 °C until further use. For WB, the thawed cells were harvested on ice using lysis buffer (1% Triton X-100, 10% glycerol, 50 mM HEPES (pH 7.4), 150 mM NaCl, 2 mM EDTA, 2 mM EGTA, and protease inhibitors) [22] and centrifuged at 13,000 rpm for 15 min. The supernatant was quantified with a bicinchoninic acid (BCA) kit and loaded onto 4–20% gels.

### 2.6. Real-Time Quantitative PCR

Total RNA was extracted in 1 mL of TRIsure (Bioline, Luckenwalde, Germany, BIO-38032) containing 20% chloroform (Acros Organics, Bridgewater, NJ, USA, 404635000). DNA contamination was eliminated from the RNA samples by processing them with a DNase I kit (Sigma-Aldrich, AMPD1). RNA was reverse-transcribed into complementary DNA (cDNA) with a First Strand DNA synthesis kit (Nzytech, Lisboa, Portugal, MB12501). The cDNA was amplified by RT–qPCR with a KAPA SYBR^®^ Fast kit (Kapa Biosystems, Cape Town, South Africa, KK4601) using the following primers from Integrated DNA Technologies: SQSTM1/p62 (FW: 5′-GGAGAAGAGCAGCTCACAGCCA-3′/RV: 5′-CCTTCAGCCCTGTGGGTCCCT-3′) and GAPDH (FW: 5′-AGCCACATCGCTGAGACA-3′/RV: 5′-GCCCAATACGACCAAATCC-3′) [23]. GAPDH gene expression was used as an endogenous control, and SQSTM1/p62 mRNA expression levels were determined by the 2(^−ΔΔCt^) ratio.

### 2.7. Binding, Endocytosis, and Trafficking of Alexa Fluor 555 EGF

HFs were seeded on cover slips in 24-well plates. After 24 h, the cells were washed with PBS and starved in binding DMEM (as described above) for 1–2 h at 37 °C. Next, the cells were kept on ice for 10 min and subsequently incubated with 100 ng/mL Alexa-EGF-555 (Invitrogen, Willow Creek Road, OR, USA, E35350) that was diluted in binding medium. After 30 min of incubation on ice, unbound ligand was removed by washing the cells with cold PBS. Time 0 was immediately fixed with 4% PFA to stop the reaction. Warm binding medium was added to the other cover slips, and the internalization of the ligand was analyzed by incubating cells at 37 °C for 5, 10, 20, 30, and 60 min followed by PFA fixation at the indicated times. Images were visualized using an Olympus IX51 inverted microscope equipped with a DP71 camera. The EGF+ vesicles were analyzed with ImageJ software.

### 2.8. Acidic Compartment Staining with LysoTracker Red

Plated cells were detached with trypsin and incubated for 15 min at 37 °C in complete DMEM containing 100 nM Lysotracker Red (LTR, Invitrogen L7528) [24]. LTR is a fluorescent probe that accumulates in acidic organelles (lysosomes and late-endosomes). The percentage of LTR^+^ fluorescence signal (*n* = 10,000 cells) was determined by flow cytometry (Beckman Coulter FC500-MPL).

### 2.9. CTSB and CTSD Activities

Cell lysates were obtained in cathepsin (CTS) buffer containing 50 mM sodium acetate (pH 5.5), 0.1 M NaCl, 1 mM EDTA, and 0.2% Triton X-100. Protein samples (1–2 μg/µL, 5 µL) were incubated at 37 °C for 90 min in 100 µL of CTS buffer supplemented with 10 μM cathepsin D and E substrate (Enzo Life Sciences, BML-P145) and 25 µM leupeptin [25]. Pepstatin A (25 µg/mL), a CTSD inhibitor, was used as a negative control. To analyze CTSB activity, 1–2 μg/µL protein samples were incubated at 37 °C for 90 min in 100 µL of CTS buffer supplemented with 20 μM CTSB substrate Z-RR-AMC (Enzo Life Sciences, BML-P137) [26]. Leupeptin, a CTSB inhibitor, was used as a negative control. The enzyme activities were quantified with a TECAN (Infinite 200 PRO) plate reader (excitation: 360 nm; emission: 440 nm) and are presented as the relative fluorescence units (RFUs)/μg protein.

### 2.10. β-Hexosamidase Activity

HFs were lysed in buffer containing 0.1% Triton X-100 and protease inhibitors. Five microliters (1–2 μg/µL protein) of sample was then incubated at 37 °C for 30 min in 100 µL of a solution containing 150 mM citrate, 0.2 M Na2HPO4 (pH 4.0) and 1.97 mM 4-methylumbelliferyl-N-acetyl-β-D-glucosaminide (Sigma-Aldrich, M2133), the substrate of β-hexosaminidase [26]. The reaction was immediately stopped by adding 190 µL of 0.3 M glycine-NaOH (pH 10.3). The β-hexosaminidase activity was measured with a TECAN infinite 200 PRO microplate reader (excitation: 360 nm; emission: 465 nm) and is represented as RFU/μg protein.

### 2.11. N-Acetylglucosaminidase Activity

The activity of β-N-acetylglucosaminidase (NAG) was determined using a colorimetric kit assay (Sigma-Aldrich, CS0780). Ten microliters of cell lysate were incubated with 90 µL of substrate solution (1 mg/mL 4-nitrophenyl-N-acetyl-β-D-glucosaminide [NP-GlcNAc] in 90 mM citrate) for 30 min at 37 °C. The hydrolysis of NP-GlcNAc releases the product p-nitrophenol. The reaction was stopped by adding 200 µL of sodium carbonate solution and incubating at 37 °C for 10 min. The absorbance of the yellow product at 405 nm was measured by the TECAN microplate reader. The experiment was performed according to the manufacturer instructions. The results are presented in U/mL.

### 2.12. Transmission Electron Microscopy (TEM)

HFs were grown on coverslips and subsequently fixed in 2% PFA (16% EM grade, Electron Microscopy Sciences, Hatfield, PA, 15710S) and 2% glutaraldehyde (TAAB Laboratory Equipment Ltd., Aldermaston, England) in 0.1 M PHEM buffer (Electron Microscopy Sciences, 11162) for 30 min at room temperature. The samples were then osmicated and further processed for resin embedding [27]. Resin blocks were sectioned (Leica Microsystems, Wetzlar, Germany; UC7), and 70 nm ultrathin serial sections were collected on formvar-coated slot grids and stained with uranyl acetate and lead citrate. The samples were observed under a transmission electron microscope (Tecnai G2 Spirit, FEI) and imaged with an Orius SC1000B charge-coupled device camera using Digital Micrograph software (both Gatan).

### 2.13. Cell Viability

Cells were stained with annexin V-FITC (Immunostep, ANXVF-200T) in PBS for 15 min at 37 °C. Propidium iodide (PI, Sigma-Aldrich, P4170, 0.1 mg/mL) [20,24] was subsequently added at the end of incubation to determine the percentage of cell death (apoptosis and/or necrosis) by flow cytometry (*n* = 10,000 events) (Beckman Coulter FC500-MPL).

### 2.14. Statistical Analysis

The collected data were analyzed using Microsoft Excel and GraphPad Prism 8.0.2. Student’s *t* test, one-way ANOVA (Tukey’s multiple comparison tests), and two-way ANOVA (Tukey’s multiple comparison tests) were used to establish significant differences between control (CTRL) and DM1 cells and differences in the treatment effects. A *p* value < 0.05 indicated statistical significance.

## 3. Results

### 3.1. Increase in Autophagy Flux and Cell Death in DM1 Cells

**Figure 1:**
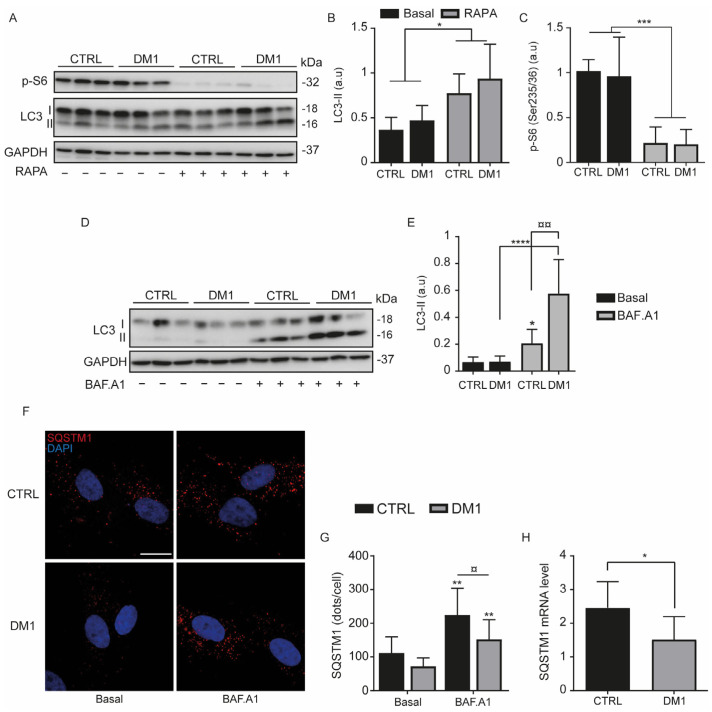
Autophagy flux is induced in myotonic dystrophy type 1 (DM1). (**A**–**E**) CTRL and DM1 cells were treated with 1 µM rapamycin (RAPA) or 100 nM bafilomycin A1 (BAF.A1) for 2 h. Expression levels of LC3-II (**A**,**B**,**D**,**E**) and p-S6(Ser235/236) (**A**,**C**) were assessed by immunoblotting and their densitometry was normalized to the loading control, GAPDH. Data are the mean ± SD of two replicates (* *p* < 0.05, *** *p* < 0.001, **** *p* < 0.0001 versus untreated cells, ¤¤ *p* < 0.01 versus BAF.A1-treated CTRL cells, with two-way ANOVA-Tukey’s test), arbitrary units (a.u). Each group (CTRL or DM1) consisted of three cell lines. (**F**,**G**) CTRL and DM1 cells were treated with BAF.A1 for 4 h. (**F**) Cells were immunolabeled with SQSTM1 antibody (Red) and nuclei stained with DAPI (blue), scale bar represents 10 μm. The original magnification is ×60. G/Quantification of SQSTM1 dots per cell was determined using ImageJ software (*n* = 100 cells/condition). Data are the mean ± SD of two replicates (** *p* < 0.01 versus untreated cells and ¤ *p* < 0.05 versus BAF.A1-treated CTRL cells, with two-way ANOVA-Tukey’s test). (**H**) Represents the SQSTM1 mRNA expression level determined by qPCR. Data are the mean ± SD of two replicates (* *p* < 0.05 respect to CTRL cells). Each group (CTRL or DM1) consisted of three cell lines. All experiments were performed at least three times.

Increased autophagy has been associated with DM1 muscle atrophy [8]; therefore, we assessed autophagy in CTRL and DM1 HFs by treating the cells with 1 µM rapamycin (RAPA) or 100 nM bafilomycin A1 (BAF. A1) for 2 h (Figure 1A–E). RAPA is an autophagy inducer that increases the autophagosome formation (LC3-II marker) via the downregulation of mTORC1, whereas BAF.A1 inhibits autophagy allowing the accumulation of LC3-II and the sequestration of substrates in the autophagosomes. RAPA (Figure 1A,B) or BAF.A1 (Figure 1D,E) significantly increased the level of LC3-II in CTRL and in DM1 HFs. Indeed, RAPA activates the formation of autophagosomes in both cell lines by significantly decreasing the phosphorylation levels of S6 (Figure 1A,C), a downstream target of mTOR. However, there were no significant differences in p-S6 levels between CTRL and DM1 cells under basal and RAPA conditions. BAF.A1 inhibits the lysosomal V-ATPase and lysosome-mediated degradation of LC3-II, thereby allowing its accumulation [28]. The difference in LC3-II between CTRL and DM1 cells was not significant under basal and RAPA conditions, but it was higher with BAF.A1 treatment (Figure 1D,E). This difference in the amount of LC3-II suggests an increase in the autophagy flux in DM1 HFs. To evaluate autophagic degradation, SQSTM1/p62 is used as an autophagy substrate due to its selective degradation [29]. In both cell lines, the analysis of SQSTM1/p62 immunostaining indicates a markedly increase in dots per cell with BAF.A1 treatment (Figure 1F,G), although this increase was lower in DM1 cells. There was also a slight reduction in SQSTM1/p62 levels between CTRL and DM1 cells under basal conditions. However, the decrease in SQSTM1 protein levels in DM1 compared with CTRL cells correlates with a diminution in SQSTM1 mRNA in the DM1 cells (Figure 1H). Therefore, the reduction in SQSTM1 protein cannot be only attributed to enhanced autophagy degradation in DM1 cells.

**Figure 2:**
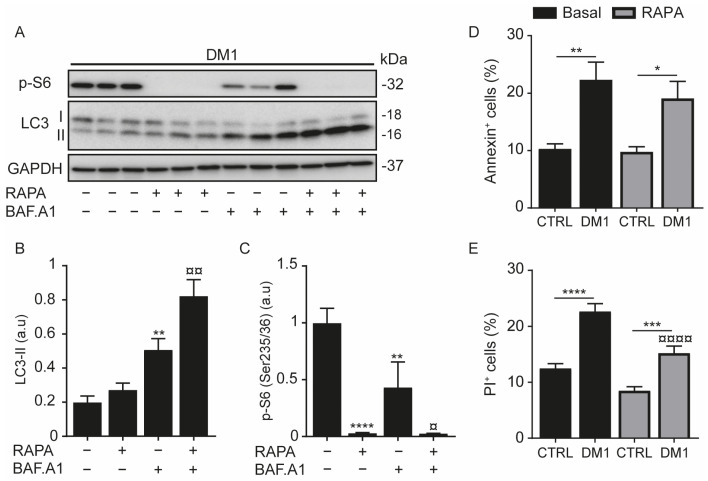
Autophagy and cell death are induced in DM1 fibroblasts. (**A**–**C**) Fibroblasts from patients with DM1 were treated with 100 nM bafilomycin A1 (BAF.A1) and/or 1 μM rapamycin (RAPA) for 2 h. Protein expression levels of LC3-II (**A**,**B**) and *p*-S6 (Ser235/236) (**A**,**C**) were determined by immunoblotting and their densitometry was normalized to the loading control, GAPDH. Data correspond to the mean ± SD of three independent lines (** *p <* 0.01, **** *p* < 0.0001 compared with untreated cells, and ¤ *p* < 0.05, ¤¤ *p* < 0.01 versus BAF.A1-treated cells with one-way ANOVA-Tukey’s test), arbitrary units (a.u). (**D**,**E**) CTRL and DM1 cells were treated with 1 µM RAPA for 24 h and then stained with annexin and propidium iodide (PI). The percentages of annexin-positive (**D**) or PI-positive (**E**) cells were detected by flow cytometry. Data are the mean % ± SEM of three replicates (* *p* < 0.05, ** *p* < 0.01, *** *p* < 0.001, **** *p* < 0.0001 with respect to CTRL cells and ¤¤¤¤ *p* < 0.0001 versus DM1 cells with two-way ANOVA-Tukey’s test), N = 10,000 events. Each group (CTRL or DM1) consisted of three cell lines. All experiments were performed at least three times.

The measuring of the autophagic flux of DM1 HFs treated with RAPA and/or BAF.A1 confirmed a significant increase in LC3-II protein levels with BAF. A1 and with the combined treatment (BAF.A1 and RAPA) (Figure 2A,B). Moreover, the difference in LC3-II levels between BAF.A1 and the combined treatment confirmed that autophagy is induced in DM1 cells. In fact, RAPA completely decreased the phosphorylation of S6 (Figure 2A,C). Increased autophagy flux in DM1, evidenced here (Figure 1D,E and Figure 2A,B) and by others, has been shown to be concomitant with an increase in apoptosis [5]. To test this event, cells were treated with RAPA for 24 h and then stained with annexin V and propidium iodide (PI). We observed that DM1 cells displayed a two-fold increase in annexin-V-positive cells (apoptosis) and PI-positive cells (necrosis) when compared with CTRL cells (Figure 2D,E). Furthermore, RAPA treatment maintained DM1-induced apoptosis (Figure 2D). Unexpectedly, it slightly decreased necrosis in DM1 (Figure 2E), although this decrease did not really reduce the significant ratio of necrotic cell death between CTRL and DM1 cells. Taken together, these data indicate that autophagy and cell death were activated in DM1-derived fibroblasts.

### 3.2. Enlarged Endosomes in DM1 Cells

**Figure 3:**
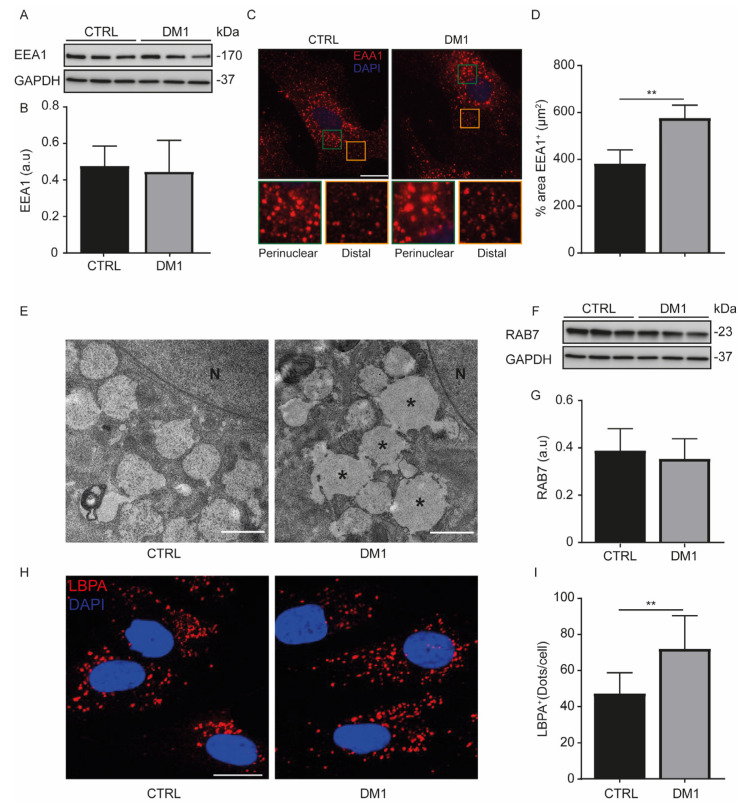
Endosomes are enlarged in DM1 cells. (**A**,**B**) Protein levels of early endosomal antigen 1 (EEA1) were determined in CTRL and DM1 cells. Densitometry is normalized to the loading control, GAPDH (a.u, arbitrary units). (**C**) CTRL and DM1 cells were immunolabeled with EEA1 antibody, and the structures were detected by confocal microscopy. Nuclei were stained with DAPI (300 nM), (original magnification ×60, scale bar is 10 µm). (**D**) The % area occupied by EEA1-positive (^+^) structures was quantified using ImageJ software (*n* = 100 cells/condition). Data are the % area ± SEM (** *p* < 0.01, with Student’s *t*-test). (**E**) Control and DM1 cells were prepared for transmission electron microscopy (TEM). Enlarged perinuclear endosomes (*) were observed in DM1 cells, as well as those of normal size. (N, nucleus; scale bar 1 μm). (**F**,**G**) Rab7 protein levels were determined and normalized to GAPDH. (**H**) CTRL and DM1 cells were immunolabeled with LBPA antibody, and the structures were detected by confocal microscopy. Nuclei were stained with DAPI (300 nM), (original magnification ×60, scale bar is 10 µm). (**I**) The number of LBPA-positive (^+^) structures per cell was quantified using ImageJ software (*n* = 100 cells/condition). Data are the number of dots ± SD (** *p* < 0.01, with Student’s *t*-test). Each group (CTRL or DM1) consisted of three cell lines. All experiments were performed at least three times, and the Student’s *t*-test was proven.

The delivery of endocytic cargo to lysosomes is initially routed to early endosomes (RAB5-positive vesicles) and then transferred to late endosomes (RAB7-positive vesicles) [30]. To better understand the endocytic degradative pathway in DM1 HFs, we first characterized the endosomes through the detection of the early endosomal antigen-1 (EEA1), a RAB5-effector that binds phosphatidylinositol-3 phosphate [PI(3)P] via its FYVE domain [31]. The EEA1 protein levels detected by Western blot were similar between CTRL and DM1 cells (Figure 3A,B). We distinguished two populations of EEA1-positive structures by immunofluorescence in both groups: a perinuclear endosomal subpopulation and a distal subpopulation (Figure 3C). Although the total number of EEA1-positive structures did not change between CTRL and DM1 cells (119 ± 12.09 vs. 120 ± 9.33, *p* = 0.67, respectively), the perinuclear early endosomes were morphologically larger in the DM1 HFs than in CTRL HFs. Indeed, the average area occupied by EEA1 structures is greater in the DM1 cells than in the CTRL cells (Figure 3D). This enlarged endosome subpopulation was also detected by transmission electron microscopy (Figure 3E). The conversion of RAB5 to RAB7 ensures the maturation of early endosomes into late endosomes [32]. The RAB7 protein levels (Figure 3F,G) did not change between cell lines, but the immunofluorescence analysis of lyso-bisphosphatidic acid (LBPA), another late endosomal marker [33], showed an increase in LBPA-positive structures in DM1 cells when compared with CTRL cells (Figure 3H,I). All these data indicate that the endosome morphology in DM1 cells is different from that of CTRL cells.

### 3.3. Enhanced Acidic Vesicles and Enzymatic Activities in DM1 Cells

**Figure 4:**
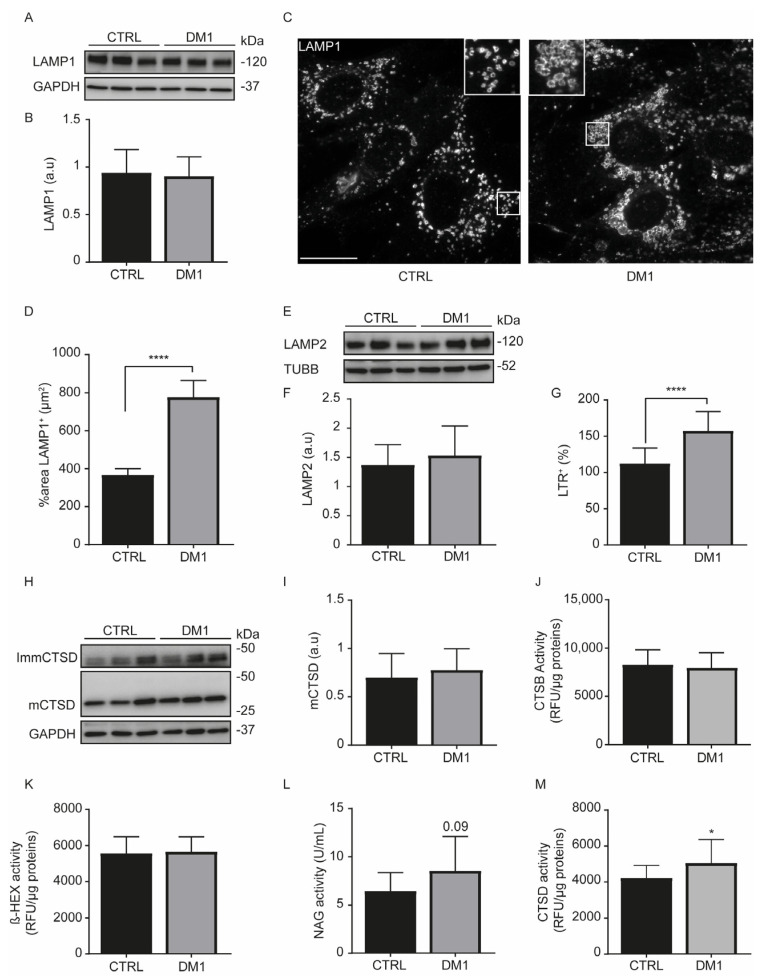
Lysosomal size and cathepsin D activity are increased in DM1 cells. (**A**,**B**) Protein expression levels of LAMP1 were detected by immunoblotting and its densitometry normalized to GAPDH, (a. u, arbitrary units). Data correspond to the mean ± SD of three replicates. (**C**) CTRL and DM1 cells were immunolabeled with LAMP1 antibody. Lysosomes were detected in human fibroblasts by confocal microscopy (original magnification ×60, scale bar is 10 µm). (**D**) The % area occupied by LAMP1 positive structures was determined with ImageJ software (*n* = 100 cells/condition). Data are the % area ± SEM (**** *p* < 0.0001, with Student’s *t*-test). (**E**,**F**) Expression levels of LAMP2 were detected by immunoblotting and its densitometry normalized to α-Tubulin (TUBB). (**G**) CTRL and DM1 cells were stained with 100 nM lysotracker red (LTR). The percentage ± SD of LTR^+^ signal was detected by flow cytometry, *n* = 10,000 events. Data are the mean ± SD of four replicates (**** *p* < 0.0001 with Student’s *t*-test). (**H**,**I**) Protein expression levels of cathepsin D (CTSD) were detected by immunoblotting. CTSD antibody detects mature (mCTSD) and immature CTSD (ImmCTSD) forms (**H**); the densitometry of mCTSD was normalized to GAPDH. Data are the mean ± SD of three replicates (**I**). (**J**–**M**) Lysosomal enzyme activities were determined by fluorimetric (**J**,**K**,**M**) or colorimetric (**L**) assays. Cathepsin B (CTSB) (**J**), β-Hexosaminidase (β-HEX) (**K**), and CTSD (**M**) activities are presented in relative fluorescence unit (RFU)/µg proteins, (* *p* < 0.05 with Student’s *t*-test). N-acetyluglucosaminidase (NAG) activity is in U/mL (**L**). Each group (CTRL or DM1) consisted of three cell lines. All experiments were performed at least three times.

Lysosomes are acidic organelles that receive material from autophagosomes and endosomes to be degraded [29]. We observed that levels of lysosomal-associated membrane protein (LAMP) 1 (Figure 4A–D) and LAMP2 (Figure 4E,F) were not significantly different between CTRL and DM1 cells. Despite the comparable expression levels of lysosomal proteins and the number of lysosomes (142.5 ± 11.78 vs. 129.8 ± 10.97, *p* = 0.43 with Student’s *t*-test), the lysosome size was increased in DM1 cells (Figure 4C), as shown by the average % area significantly occupied by LAMP1^+^ structures (Figure 4D). Consequently, we stained cells with LTR, a fluorescent probe that selectively accumulates in acidic vesicles, especially in lysosomes and in late endosomes [34]. Interestingly, there were significantly more LTR^+^ signals in the DM1 cells (Figure 4G), as previously demonstrated in the DM1 fly model and in DM1 myoblasts [5]. We think that the increment in LTR signal is related to the increased lysosomal size in DM1 cells. Lysosomal enzymes are essential for the degradation of lysosomal cargoes, and an increased expression of cathepsin B (CTSB) protein level has been reported in human DM1-NSCs [6]. We investigated whether there were differences in the levels of cathepsin (CTS) enzymes between CTRL and DM1 cells. Pro and intermediate enzyme forms were classified as immature CTS. Variations in CTS protein levels were clearly observed within each group of cell line; however, the mature forms of CTSB (Figure S1A,B), CTSC (Figure S1D,E), CTSL (Figure S1G,H), and CTSD (Figure 4H,I) were not different between the two cell types. A similar result was observed when determining the mCTS/immature ratio (Figure S1C,F,I). When measuring the enzyme activities, CTSB (Figure 4J) and β-HEX (Figure 4K) levels were not different between the two cell types. However, the NAG (Figure 4L) and CTSD (Figure 4M) activities were increased in DM1 cells when compared with CTRL cells; similar results were observed in dystrophic muscles [35]. Therefore, DM1-derived fibroblasts exhibited increased lysosomal size and a significant increase in CTSD activity, suggesting a degree of lysosome functionality.

### 3.4. Defect in Fluorescent EGF Binding and Delay of EGFR Degradation in DM1 Cells

**Figure 5:**
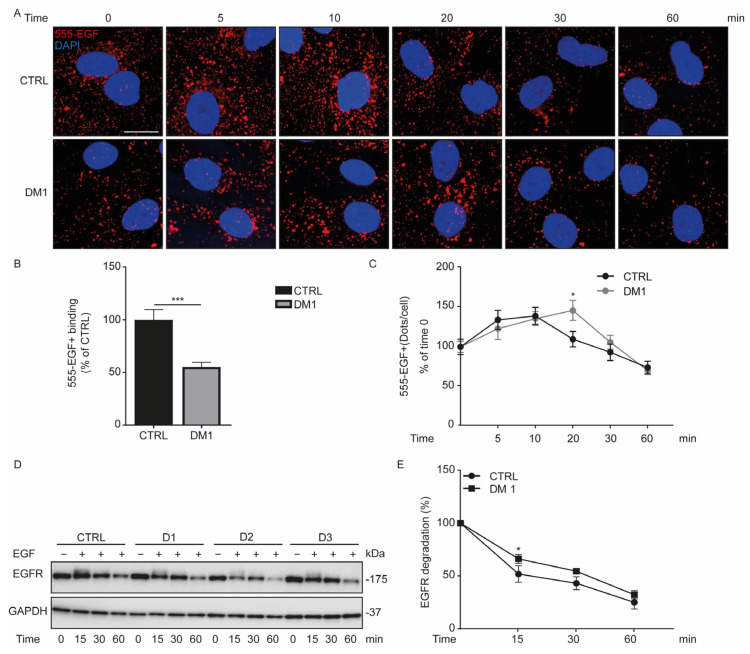
EGFR internalization decreases in DM1 cells. (**A**–**C**) Serum-starved CTRL and DM1 cells were incubated with 100 ng/mL Alexa-555-EGF (555-EGF) for 30 min on ice. After washing, cells were incubated at 37 °C for the indicated times (0, 5, 10, 20, 30, and 60 min) and then fixed. (**A**) Representative images of the fluorescent EGF (555-EGF), nuclei were stained with 300 nM DAPI (scale bar is 10 μm; original magnification is ×60). (**B**) Data are the percentage of 555-EGF^+^ dots ± SEM per cell at time zero (*** *p* < 0.001 with Student’s *t*-test), reflecting the binding of the ligand. (**C**) Data are the percentage ± SEM of 555-EGF^+^ relative to time zero (* *p* < 0.05 DM1 versus CTRL cells at 20 min, with a multiple Student’s *t*-test), reflecting the trafficking and degradation of the fluorescent EGF. (**D**,**E**) CTRL and DM1 were starved overnight and then incubated at different time points (0, 15, 30, and 60 min) with 100 ng/mL EGF. The EGFR protein level was normalized to GAPDH, and the percentage was determined relative to time zero. (**E**) Data are the mean percentage ± SD of EGFR degradation at the indicated times (* *p* < 0.05, with multiple Student’s *t*-tests).

Endosomes (Figure 3C) and lysosomes (Figure 4C) are enlarged in DM1 cells when compared with CTRL cells; therefore, we examined their impact in the degradative endocytic pathway by incubating fibroblasts with Alexa-555-EGF (555-EGF) for 30 min on ice and followed the evolution of endocytosed 555-EGF by immunofluorescence microscopy at different time points (Figure 5A). At time zero, 555-EGF binding was significantly reduced in DM1 cells compared with CTRL cells (Figure 5B). During the incubation of the fibroblasts at 37 °C, the amount of 555-EGF was quantified at different times from 5 to 60 min. Given the difference in 555-EGF binding, the percentage of 555-EGF^+^ in each group was considered to be 100 at time zero. Firstly, we observed an increase in 555-EGF^+^ vesicles in CTRL and DM1 HFs at 10 and 20 min, respectively (Figure 5C). Subsequently, the amount of endocytosed 555-EGF begin to decrease and we observed a significant delay in 555-EGF degradation in DM1 cells compared with CTRL at 20 min. Such a delay disappeared around 60 min of incubation in DM1 cells. We then conclude that there was a decrease in the fluorescent EGF binding to EGFR in DM1, followed by a delay in 555-EGF trafficking and degradation during the first 20 and 30 min of endocytosis.

To corroborate the defect in the endocytosis trafficking of 555-EGF in DM1 cells, we assessed the EGFR protein level in HFs upon EGF ligand stimulation. Serum-starved cells were treated with 100 ng/mL EGF for 15, 30, and 60 min (Figure 5D). EGF treatment stimulates and triggers the activation and internalization of the EGF receptor (EGFR) via the endocytic pathway. At time zero, the level of EGFR protein between CTRL and DM1 cells was similar (100 ± 15.81 vs. 101.44 ± 32.44, respectively). Nevertheless, the percentage of EGFR in each group was considered to be 100 at time zero. This percentage started to decrease progressively in CTRL and DM1 cells from 15 min to 60 min (Figure 5E). Additionally, the degradation of EGFR was slower in DM1 cells than in CTRL cells at 15 min (*p* = 0.02). Interestingly, this difference in EGFR protein level tended to disappear from 30 min (*p* = 0.07) and become similar at 60 min in CTRL cells and in DM1 cells. Overall, these results sustain a delay in EGF-stimulated EGFR degradation in DM1 cells between 0 and 30 min.

### 3.5. Activation of EGFR Signaling Pathway upon EGF Stimulation in DM1 Cells

**Figure 6:**
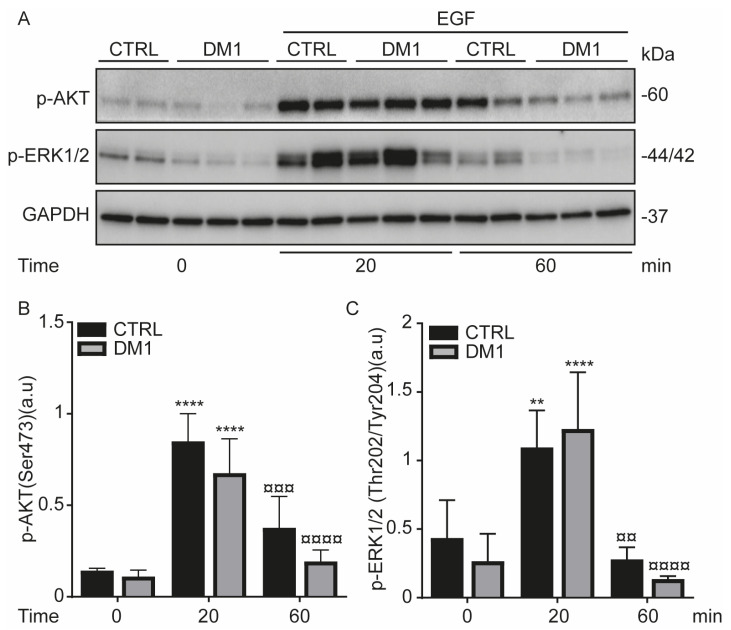
EGF-induced EGFR activation increases AKT/ERK signaling in DM1. (**A**–**C**) Serum-starved CTRL and DM1 cells were stimulated with 100 ng/mL EGF for 20 min and 60 min. Densitometries of p-AKT (**A**,**B**) and p-ERK1/2 (**A**,**C**) were normalized to GAPDH. Values are the mean ± SD of two replicates (** *p* < 0.01, **** *p* < 0.0001 versus time zero and ¤¤ *p* < 0.01, ¤¤¤ *p* < 0.001, ¤¤¤¤ *p* < 0.0001 versus 20 min with two-way ANOVA-Tukey’s test).

EGF-induced EGFR activation stimulates downstream signaling pathways such as AKT and ERK1/2 [36], which results in an increase in their phosphorylation levels and EGFR endocytosis [37]. To study whether EGFR signaling is activated in DM1 cells, serum-starved cells were treated with EGF for 0, 20, and 60 min. Upon EGF stimulation, the phosphorylation levels of AKT (Ser473) and ERK1/2(Thr202/Tyr204) significantly increased in both cell lines at 20 min (Figure 6). This increase was markedly reduced at 60 min, suggesting that the phosphorylation observed at 20 min was followed by a phase of decay, which results in an attenuation of the EGFR signaling. It is important to note that despite the decrease in EGF binding to EGFR, the EGFR downstream signaling was activated at 20 min in DM1 cells as compared with time zero. However, there was a sustained decrease in p-AKT in DM1 cells as compared with CTRL cells at 20 and 60 min. These data allow us to conclude that EGF-activated EGFR is able to signal from endosomes and its delay in the endocytic trafficking does not affect the downstream (AKT and ERK1/2) signaling pathways in DM1 cells, but highlights a difference in p-AKT levels between cell lines.

### 3.6. Endocytosed EGFR Is Sorted and Degraded into Lysosomes in DM1 Cells

**Figure 7:**
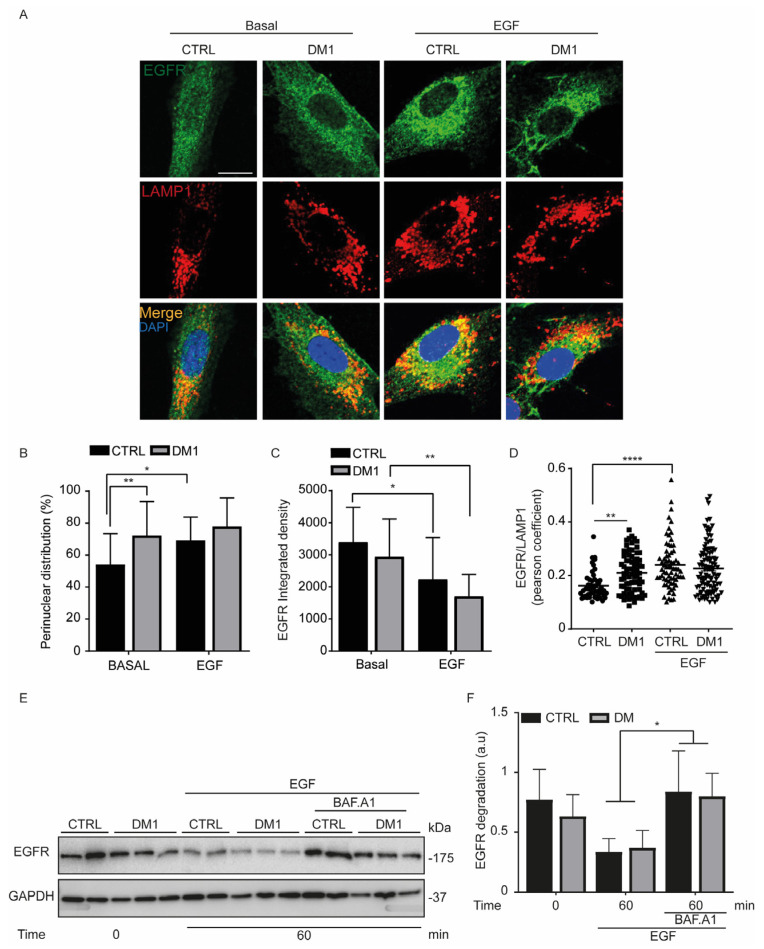
EGF-induced EGFR is sorted into lysosomes. (**A**–**D**) Serum-starved cells were incubated with 100 ng/mL EGF for one hour. (**A**) Cells were co-labeled with EGFR and LAMP1 antibodies. Nuclei were stained with 300 nM DAPI (scale bar is 10 μm; original magnification is ×60). (**B**) The perinuclear distribution of EGFR that was determined by manual counting, *n* = 80–100 cells (* *p* < 0.05, ** *p* < 0.01 two-way ANOVA Tukey’s test). (**C**) The fluorescence intensity of EGFR represented as integrated density from ImageJ software (** *p* < 0.01, **** *p* < 0.0001 two-way ANOVA Tukey’s test). (**D**) Reflects the co-localization between EGFR/LAMP1 using Pearson’s coefficient (** *p* < 0.01, **** *p* < 0.0001 with two-way ANOVA). All experiments were performed at least two times. (**E**,**F**) Serum-starved cells (CTRL and DM1) were pre-treated for 60 min with 100 nM BAF.A1 and then stimulated with 100 ng/mL EGF for one hour. (**F**) Represents the level of EGF-induced EGFR degradation normalized to GAPDH. Data are the mean ± SD of two replicates, (* *p* < 0.05, with two-way ANOVA Tukey’s test).

To determine whether EGF-stimulated EGFR is sorted to lysosomes, serum-starved cells were treated with EGF (100 ng/mL) and co-labeled with EGFR and LAMP1 antibodies (Figure 7A). EGFR was significantly perinuclear in unstimulated DM1 cells (Figure 7A,B). This result can explain, at least in part, the lower binding of EGF to EGFR in DM1 cells compared with the control cells. This perinuclear distribution persisted and was unchanged upon EGF treatment in DM1 cells, unlike in CTRL cells. In agreement with this intracellular distribution of EGFR, the fluorescence intensity of EGFR significantly decreased upon EGF stimulation for 60 min in CTRL and DM1 cells (Figure 7C). We then evaluated the colocalization of EGFR and LAMP1. Despite the significant difference between CTRL and DM1 cells under basal conditions, the colocalization of EGF-stimulated EGFR with LAMP1 was similar in both cell types at 60 min (Figure 7D). Subsequently, serum-starved cells were pretreated with the lysosomal inhibitor BAF.A1 (100 nM) for one hour and then stimulated for another hour with EGF. A significant accumulation of EGFR was observed in the BAF.A1-treated cells compared with EGF alone (Figure 7E,F), indicating that BAF.A1 reverses EGF-induced EGFR degradation in CTRL and in DM1 cells. Taken together, these results indicate that the degradation of EGF-stimulated EGFR occurs via the endosomal–lysosomal pathway in both cell lines, although the binding of EGF to its receptor is reduced in DM1 cells.

## 4. Discussion

Human fibroblasts from DM1 patients displayed an increase in autophagic flux, despite the downregulated *SQSTM1* expression. Most important, lysosomal enzyme activities such as CTSD and NAG were augmented. Moreover, DM1 cells exhibited enlarged perinuclear endosomes and an increase in lysosomal size, which was in correlation with the significant increase in LTR fluorescence signal. Additionally, EGF-stimulated EGFR was internalized and degraded in lysosomes, and such a degradation was prevented with BAF.A1 treatment in CTRL and DM1 cells. However, the binding of EGF to EGFR and EGFR trafficking were significantly reduced in DM1 cells. In spite of the activation of the EGFR signaling pathway, EGFR endocytosis was slowed in DM1 cells.

Autophagy was upregulated in DM1-derived fibroblasts (Figure 1), and the impairment of this catabolic process has been widely reported in different DM1 models [5,6,7,9]. However, fibroblasts from three investigated DM1 patients differentiated into myotubes displayed a blocked autophagic flux under the combination of chloroquine treatment and/or starvation [9]. At the same time, chloroquine did not generate additional autophagic vacuoles over the already induced basal autophagy in DM1-NSCs [6]. In our models, LC3-II (an indicator of autophagosome formation) was significantly accumulated with BAF.A1 treatment (Figure 1D,E), which was accompanied by an increase in SQSTM1/p62 puncta (Figure 1F,G). Autophagy is negatively regulated by the Class I PI3K/AKT/mTOR pathway [38], and its exacerbation contributes to muscle atrophy. In muscle atrophy [10] and eventually in DM1, the phosphorylation level of AKT is downregulated and correlated with the reduction in DMPK and muscleblind-like 1 (MBNL1) proteins [16]. Rapamycin, an mTOR inhibitor, promoted LC3 lipidation in the DM1-derived fibroblasts (Figure 1A,B) but not in muscle tissue from HSA^LR^ mice [9]; however, it was reported to sufficiently improve muscle function. Hence, this effect was attributed to an mTOR-independent mechanism. In our study, rapamycin downregulated S6(Ser235/236) phosphorylation levels (Figure 2A,C) and did not reduce apoptosis in DM1 HFs after 24 h (Figure 2D). Consistent with these data, autophagy inhibition through *Tor* overexpression prevents CTG-induced muscle atrophy in DM1 model flies [5]. Another study reinforced that autophagy inhibition improved the proliferation of DM1 skeletal muscle satellite cells via the overexpression of MBNL1, which activates mTOR [39]. Although autophagy outcomes have been controversial among the studied DM1 models, there is evidence that AKT/mTOR pathway dysregulation [5,6,9] is involved in the pathogenesis of myotonic dystrophy.

AKT phosphorylation is also regulated by the activation of tyrosine kinase receptors, such as the EGFR. Upon stimulation, EGFR triggers a downstream signaling cascade that involves ERK1/2 and AKT. The internalization and degradation of EGFR in lysosomes promotes signal attenuation [12]. To date, there are not sufficient data to fully clarify the role of endocytosis in DM1. Increased endocytosis was associated with dystrophic muscle fibers, as evidenced by the formation of vesicles from transverse (T) tubules [35,40]. T-tubules are essential for the coordination of calcium release and muscle contraction. Endocytosis ensures the formation of T-tubules from the sarcolemma by membrane sequestration and stabilizes its integrity through tight junctions with the sarcoplasmic reticulum [41]. Therefore, T-tubule alterations in DM1 [42,43] allow us to think that endocytosis may be altered in skeletal muscles cells. In this study, we demonstrated that DM1 HFs contained enlarged endosomes (Figure 3C–E) and lysosomes (Figure 4C,D). This phenotype suggested that the endocytosis pathway could be altered. Interestingly, the binding of EGF to EGFR was reduced and the receptor internalization was also decreased (Figure 5). Despite the enlarged perinuclear endosomes in DM1 cells, the endocytic trafficking was slowed during early EGFR trafficking, but it subsequently reached the EGFR level of the CTRL cells in the later phase of EGF stimulation (e.g., 1 h). A previous study attributed the decrease in EGFR degradation to complete AKT inhibition leading to an accumulation of non-degraded EGF–EGFR complex in EEA1-positive structures [12]. Accordingly, the decrease in AKT phosphorylation [16] could explain the endosome morphology in DM1 cells. Additionally, a crosstalk between autophagy and the endocytic pathway has been established, because autophagy inhibition has been shown to induce damaged endosomes, reduce EGFR recycling, and prevent EGF-mediated signaling [44]. The induction of autophagy in DM1 cells allows us to think that endosomes are not damaged in our experimental model. In addition, there was no co-localization between EEA1 and LAMP1 (data not shown).

On the other hand, EGFR endocytosis depends on the expression and localization of the EGFR receptor and on its binding to the ligand [37]. We have not observed a significant difference in EGFR protein levels between cell lines, although it fluctuated in serum-starved CTRL and DM1 cells. In both cell lines, the EGFR-bound EGF resulted in a significant activation of AKT and ERK1/2, which was subsequently down-modulated (Figure 6), suggesting a degradation of EGFR [45]. Therefore, primary human fibroblasts are a good model to study EGFR-mediated signaling. We hypothesized that the decrease in EGF binding might delay the internalization of the receptor in DM1 cells but did not impede its sorting and its degradation in lysosomes, which was clearly prevented with BAF.A1 pre-treatment (Figure 7E,F). Similar results were expected with the addition of 50 µM leupeptin, but without success. This may be due to inadequate concentrations or very short treatment duration. Even though EGFR endocytosis was delayed in DM1 cells, we think that the significant increase in LBPA proteins and CTSD activity might be a compensatory mechanism that mediates the sorting and the degradation of EGF–EGFR complexes.

We conclude that the delay in EGFR trafficking is not due to decreased receptor expression or inhibition of AKT in EGF-treated DM1-derived fibroblasts. However, it is the consequence of a perinuclear distribution of the receptor, which significantly reduces the binding of the ligand. Therefore, it will be interesting to stimulate DM1 cells with low EGF concentrations to promote EGFR recycling and to check whether the perinuclear distribution of EGFR will decrease and the ligand binding rate will be restored. The insulin receptor is a tyrosine kinase receptor which is endocytosed upon insulin binding to regulate metabolism [46]. Low insulin responsiveness has been attributed to the aberrant alternative splicing of insulin receptor in DM1 muscle cells [47]. Although descriptive, we think that our findings will provide new insights into the low-insulin-induced metabolic effects in DM1. Moreover, a subcellular distribution of insulin receptor might be a contributing factor to insulin resistance [46]. Nevertheless, further studies are required to fully elucidate EGFR activity in DM1, because altered EGFR signaling is associated with human cancers [48]. Several publications have indexed the overall risk of cancers in DM1 patients [4,49], with the most prevalent being skin cancers, specifically basal cell carcinoma [50,51]. To that end, we cannot exclude endocytic pathway alterations from the mechanism underlying the onset of cancers and/or metabolic diseases [52] in DM1 patients. This study was performed in DM1 primary human fibroblasts; therefore, it would be essential to elucidate the role of endocytosis in DM1 skeletal muscle cells. Impaired endocytosis could be involved in the etiology of DM1 by altering calcium homeostasis. This hypothesis seems to be supported by several studies that have reported a morphological alteration of the sarcoplasmic reticulum and/or the tubular system in myotonic dystrophy [42,43].

## Data Availability

Not applicable.

## Supplementary Materials

The following supporting information can be downloaded at: https://www.mdpi.com/article/10.3390/cells11193018/s1, Figure S1: Cathepsin B, C, and L proteins are not modulated in DM1 fibroblasts.
