# Supplementary material for: Delay of EGF-Stimulated EGFR Degradation in Myotonic Dystrophy Type 1 (DM1)
Source: Cells. 2022 Sep 27;11(19):3018. doi: 10.3390/cells11193018 (PMC9562898; doi:10.3390/cells11193018)
Supplement: Supplementary file 1 [file cells-11-03018-s001.zip › cells-1833508-supplementary.pdf]

cells MDPI

Supplementary

Delay of EGF-Stimulated EGFR Degradation in Myotonic Dystrophy Type 1 (DM1)

Eva Alegre-Cortés [1,3], Alberto Giménez-Bejarano [1,3], Elisabet Uribe-Carretero [1,2,3], Marta Paredes-Barquero [1,3], André R. A. Marques [4], Mafalda Lopes-da-Silva [4], Otília V. Vieira [4], Saray Canales-Cortés [1], Pedro J. Camello [5,6], Guadalupe Martínez-Chacón [1,2,3], Ana Aiastui [2,7,8], Roberto Fernández-Torrón [2,8,9,10,11], Adolfo López de Munain [2,8,9,10,11], Patricia Gomez-Suaga [1,3], Mireia Niso-Santano [1,2,3], Rosa A. González-Polo [1,2,3], José M. Fuentes [1,2,3,*] and Sokhna M. S. Yakhine-Diop [2,3,*]

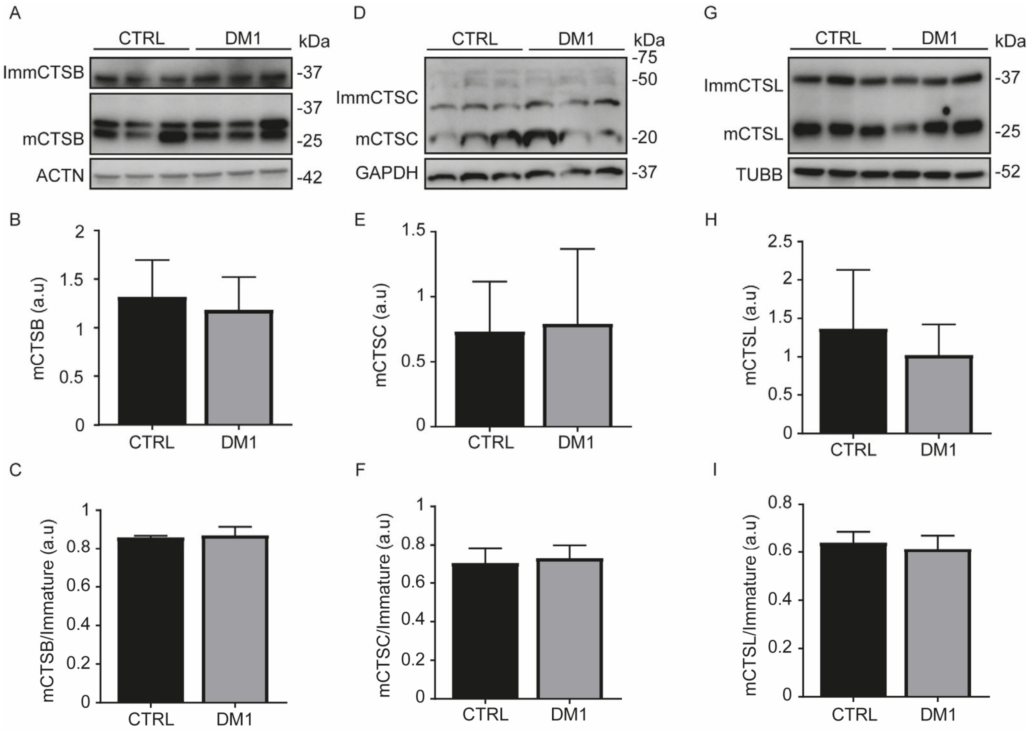

Figure S1. Cathepsin B, C and L protein are not modulated in DM1 fibroblasts. Antibodies of Cathepsin B (CTSB), CTSC, CTSL detect mature cathepsin (mCTS) and immature (Imm) cathepsin (pro and intermediate) isoforms of the respective cathepsin proteins. There were no significant differences in the expression of cathepsin either determined as mature CTS normalized to the respective loading controls actin (ACTN), GAPDH, and α-tubulin (TUBB) (A, B; D, E; and G, H) or mature CTS normalized to immature CTS (C, F, and I). Data are the mean ± SD of three replicates. Each group (CTRL or DM1) consisted of three cell lines. Experiments were done at least three times. Student's t-test was applied.
